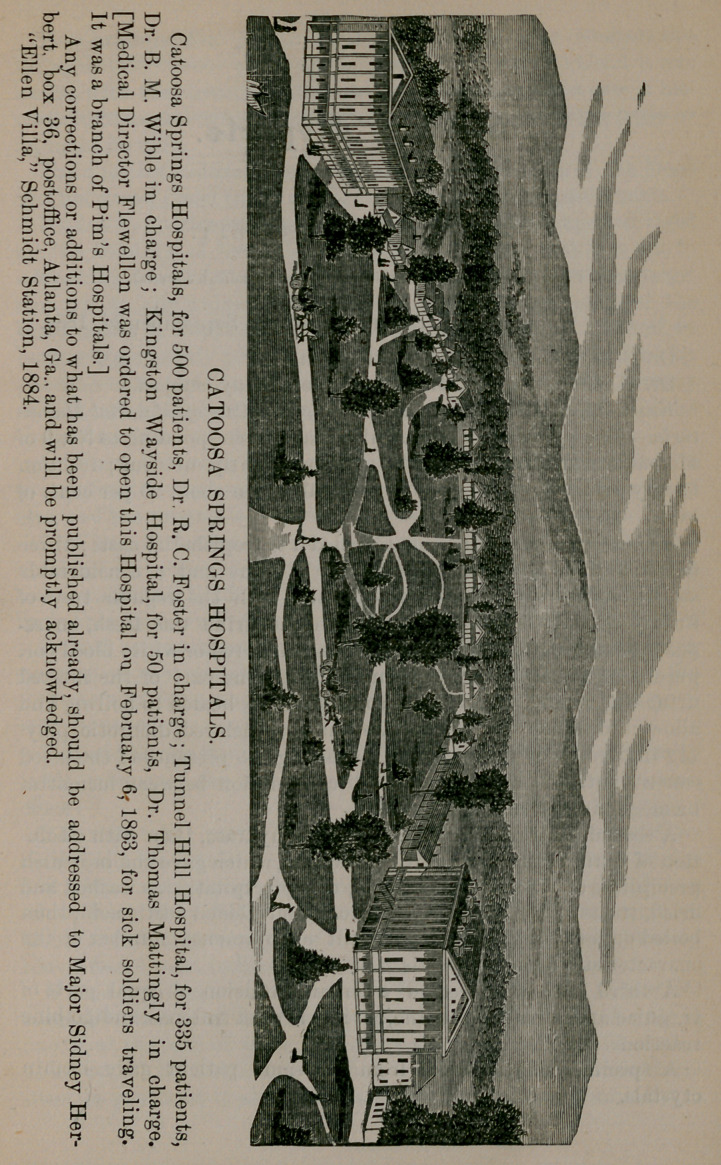# Confederate Surgeons

**Published:** 1884-10

**Authors:** Sidney Herbert


### CONFEDERATE SURGEONS.

Roster of Surgeons with Georgia Troops—Hospitals and their Officials—From Records of Medical Directors A. J. Foard and E. A. Flewellen.

### GEORGIA CONFEDERATE HOPITALS—ARTICLE SECOND.

By SIDNEY HERBERT.

The record books of Medical Director A. J. Foard, of the Army of the Tennessee, show the following named General Hospitals to have been located in Georgia:

#### ATLANTA HOSPITALS.—DR. JOS. P. LOGAN, POST SURGEON.

Empire Hospital, for 250 patients, Dr. W. P. Hardin in charge.

Fair Grounds Hospital (No. 1), for 400 patients, Dr. W. H. Brown in charge.

Fair Grounds Hospital (No. 2), for 400 patients, Dr. Robert Battey in charge.

Gate City Hospital, for 400 patients, Dr. Paul F. Eve in charge.
Grant Hospital, for 100 patients, Dr. J. C. Mullens in charge.
Medical College Hospital, for 200 patients, Dr. W. F. Westmoreland in charge.

ROME HOSPITALS.—DR. L. T. PIM, POST SURGEON.

Bell Hospital, for 225 patients, Dr. W. L. Nicholls in charge.
Lumpkin Hospital, for 229 patients, Dr. E. McDonald in charge.
Pim Hospital, for —— patients, Dr. W. C. Nichols in charge.
Polk Hospital, for —— patients, Dr. L. C. Pynchon in charge.
Quintard Hospital, for 206 patients L. F. Richberg in charge.

DALTON HOSPITALS.—DR. F. H. EVANS, POST SURGEON.

Cannon Hospital, for 200 patients, Dr. D H. Morrison in charge
Oliver Hospital, for 250 patients, Dr. J. M. Henson in charge.
St. Mary's Hospital, for 250 patients, Dr. W. J. Holt in charge.

RINGGOLD HOSPITALS.—DR. C. B. GAMBLE, POST SURGEON.

Bragg Hospital, for 300 patients, Dr. G. E. Redwood in charge.
Buckner Hospital, for 200 patients, Dr. W. T. McAllister in charge.
Foard Hospital, for 200 patients, Dr. G. W. Curry in charge.

[Of the above named hospitals there is but little on record. The "Empire" was in the old "Empire block," on Whitehall street, between Hunter and Mitchell streets. The old American Hotel, where Block's candy factory now stands, corner of Alabama and Pryor streets, was, I think, the "Gate City." The Quintard Hospital, at Rome, was named for the Rt. Rev. C. T. Quintard, now Bishop of Tennessee; the "Polk," for Lieut-Gen. (and Bishop) Leonidas Polk, the "Pim," for the Post Surgeon; the "Bragg," at Ringgold, for General Braxton Bragg; the "Buckner," for Gen. S. B. Buckner; the "Foard," for Medical Director A. J. Foard. There was also a Foard Hospital in Chattanooga named in his honor.]

HOSPITAL STEWARDS.

Burke, Patrick H., at Buckner Hospital, in Ringgold, October 17, 1862.

Cavins, George A., at Catoosa Springs Hospital, January 7, 1862.

Cox, J. T., at Buckner Hospital, in Ringgold, February 7, 1863.

Fox, Amos, Atlanta, September 11, 1862.

Hawks, C. L., Empire Hospital, in Atlanta, March 15, 1863.

Hays, Hosea B., at Bragg Hospital in Ringgold, December 19, 1862.

McMath (or Nath), J. E., at Grant Hospital, in Atlanta, July 25, 1862.

Sawtelle, J. Y., at Medical College Hospital in Atlanta, October 2, 1862.

Smith, S. F., at Post Surgeon's office in Atlanta, Sept. 2, 1862.

Sneed, W. J., at Foard Hospital in Ringgold, February 24, 1863.

Stanton, J. N., at Fair Grounds Hospital in Atlanta, October 16, 1861.

Sykes, R. L., at Oliver Hospital in Dalton, October 27, 1862.

This completes the transcript from the record books of Medical Directors, Foard and Flewellen, so far as these books go. Of course there are other books and records, and from them I hope to gather further official information.

Dr. H. F. Campbell, of Augusta, was in charge of the Georgia Hospitals in Richmond, Va., and from him and Dr. J. P. Logan, of Atlanta, and Dr. S. H. Stout, now of Texas, as well as Medical Director E. A. Flewellen, at present General Manager of a railroad at Opelika, Ala., valuable information will be sought for future articles.

## Figures and Tables

**Figure f1:**
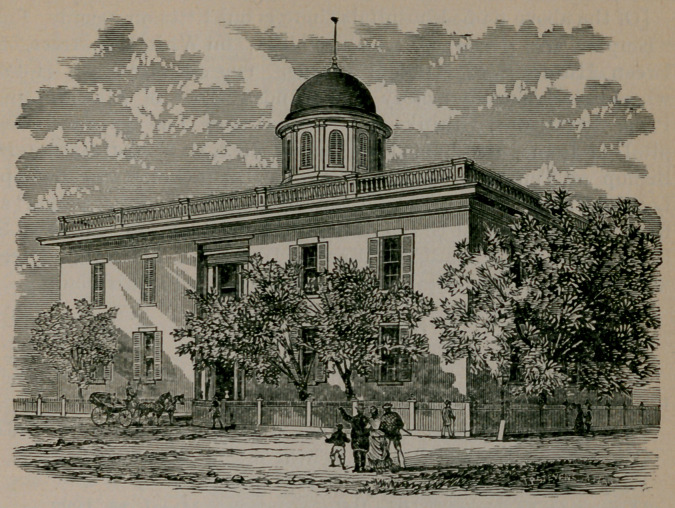


**Figure f2:**